# A carbon-monitoring strategy through near-real–time data and space technology

**DOI:** 10.1016/j.xinn.2022.100346

**Published:** 2022-11-03

**Authors:** Zhu Liu, Zhu Deng, Xiaoting Huang

**Affiliations:** 1 Department of Earth System Science, Tsinghua University, Beijing 100084, China; 2 Product and Solution & Website Business Unit, Alibaba Cloud, Hangzhou 311121, China

**Keywords:** carbon emission

## Abstract

In this perspective, we proposed an innovative strategy that coupled near-real-time emission data with satellite observations to make a reliable and precise global carbon-monitoring system.

## Main text

With nations pledging net-zero emissions by 2050, tracking dynamics of anthropogenic carbon emissions is proving to be essential for global climate change governance and carbon neutrality achievement. Critical challenges remain in the timely, precise, and robust monitoring of carbon emissions given that the anthropogenic carbon emissions are estimated by the emission accounting inventories (e.g., emission datasets of IEA) that are based on energy statistics rather than measured directly. These “bottom-up” estimates, which are often related to incompleteness of energy statistics, uncertainty in emission factors, and inconsistencies in data quality between regions, highlight the urgent need for technology and research paradigm innovation.

On the other hand, the “top-down” space observation technologies (satellite and its integrated observation system) provide a potential measurement and verification solution that has been rapidly developed, taking advantage of its real-time, continuous, large-scale, and repeated observations. Europe (SCIAMACHY), Japan (GOSAT 1/2), the United States (OCO2/3), Canada (GHGSat-D), and China (TanSat/Gaofen-5) have successively launched satellites with CO_2_ concentration observation capability in the last two decades.

However, gaps remain in tracking anthropogenic emissions from space independently for several CO_2_ features and satellite limitations:^1^ (1) the satellites measure column-averaged dry air mole fraction of CO_2_ (XCO_2_) instead of CO_2_ emissions, which means conversion is needed to trace the enhancement in XCO_2_ from human-related CO_2_ emissions. (2) Signals from regional anthropogenic emissions are much smaller than the concentration changes from atmospheric interannual variability and transportation. Thus, the background fluctuations masking human signals are still a frontier to attribute signal changes to anthropogenic emissions. (3) As the satellite observations operated through a “pinpointing” scanning measure, there is always a trade-off between scope and resolution, as coverage of large emission sources can only be obtained by reducing the temporal resolution restricted by narrow swaths of spaceborne sensors. In other words, achieving frequent revisits or tracking emissions globally and contemporaneously undoubtedly requires a constellation.

**Figure 1:**
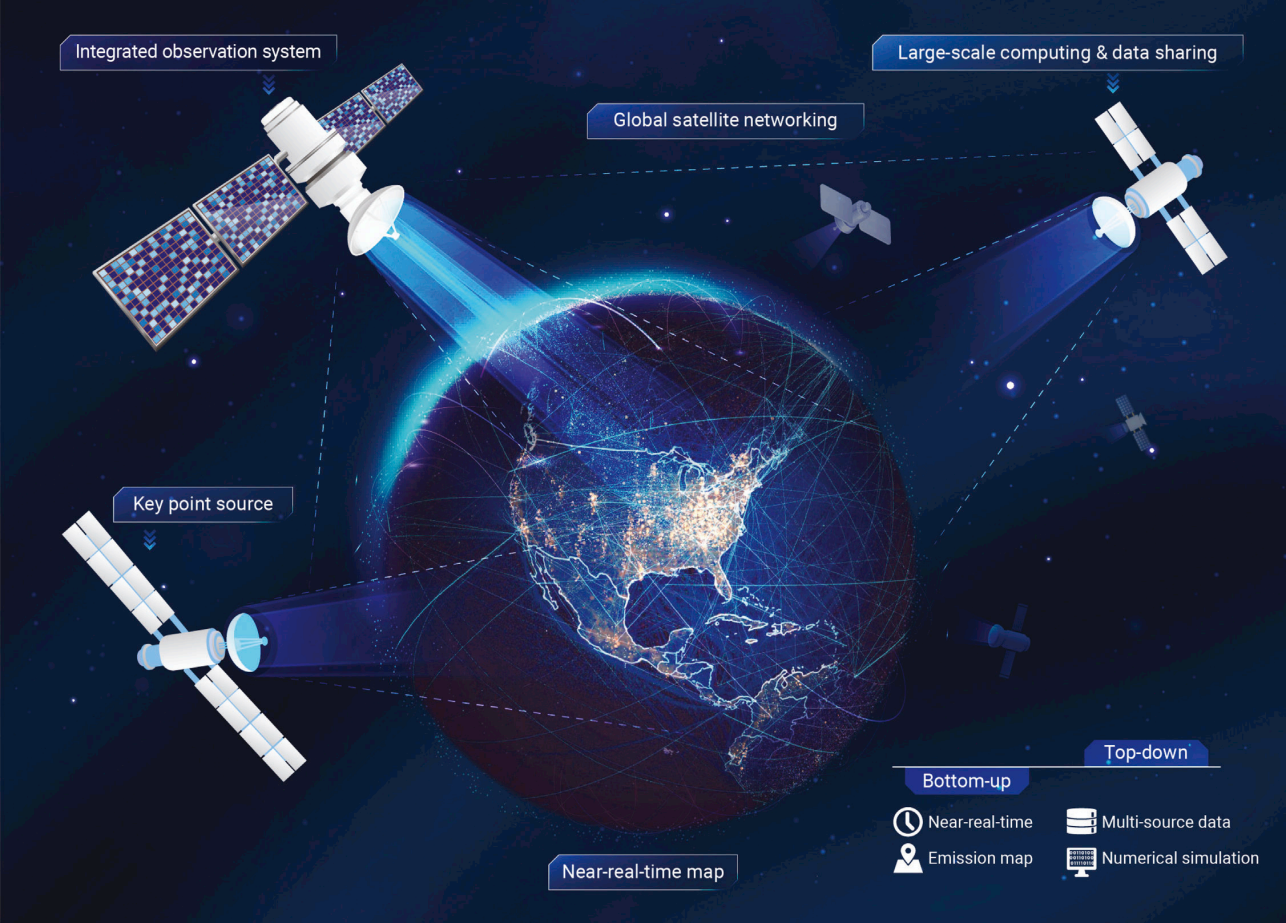
Framework of the carbon-monitoring strategy

Here, we propose a new strategy (Figure 1) that has the potential to be able to monitor global carbon emissions based on the near-real–time emission dataset and the integration, networking, and assimilation of a large number of satellites of different origins. The strategy will first draw a global high spatial–temporal resolution emission map based on near-real–time data and then allow satellites to target by scanning the large emitter. Finally, with more and more data and satellites available, such space CO_2_ monitoring systems will achieve the coverage, resolution, and timeliness that are required for concrete global emission monitoring. The strategy is compromised of three components.

## Drawing the near-real–time global emission map

The current challenge to coupling the “bottom-up” emission inventory with “top-down” satellite observation is that the inventory cannot match the observation in both the spatial and temporal dimensions.^2^ The inventory usually lacks high temporal resolution, with only annual or monthly data available, and has a time lag of at least 1 year. Recent progress on the emission estimates based on near-real–time human activities data (smart meters, grid data, transportation mobility, etc.) provides opportunities to advance the emission estimates into near real time.^3^^,^^4^^,^^5^ By using geographic information signals, grid data, or *in situ* observations (e.g., NOx) as CO_2_ emission proxies, near-real–time daily CO_2_ emission maps can be developed^6^ to serve as prior information to facilitate the detection of potential point-source emitters and validation of satellite observations.^7^ The near-real–time global emission maps display high-resolution spatial information on carbon emissions anywhere and anytime when satellites visit (daily scale), thus providing a credible baseline map for satellite observations.

## Advancing key point-source monitoring by satellite

Key point emission sources (cities, power plants, etc.) are the main source of anthropogenic carbon emissions (e.g., cities contribute 70% of anthropogenic emissions). With the near-real–time global emission map being constructed, the satellites could now only target huge emitters like cities and plants. This will help keep satellites’ advantages on their high resolution and timeliness without humbling the whole emission coverage. For example, the TROPOspheric Monitoring Instrument with km-level spatial resolution is adequate for detecting extreme methane leakage from accidental blowouts and methane ultra-emitters.^8^ Such satellites measure radiances in the spectral bands (both visible bands and near-infrared bands) and detect carbon dioxide concentrations based on molecular absorption spectra. In combination with other information (ground observations, meteorological data, etc.), atmospheric inversion models can convert the observed CO_2_ concentration values over the key point sources back to the original concentration field and estimate their carbon emissions. For example, current studies that used Gaussian plume model and OCO-2/3 XCO_2_ retrievals to estimate corresponding cross-sectional CO_2_ fluxes from large emission sources showed broad consistency between satellite-based results and emission inventories.^9^ Continuously and accurately tracking emissions on large emission sources could provide a solid basis for large-scale satellite constellation observations.

Future developments can be made in several aspects. (1) Satellites with diverse attributes can work together to meet different requirements by combining satellites with multiple spatial and spectral resolutions to track plumes at different scales. For global observation, satellites such as GOSAT with sparse but global coverage, a 3-day revisit period, and a continuous data stream target global observations. As for point-source observation, GHGSat with high sensitivity but spatial coverage limit or multispectral instruments such as Sentinel-2 and WorldView-3 with the high spatial resolution is capable of factory-level monitoring. (2) With more advanced satellite missions and their more sensitive instruments to observe XCO_2_ and XCH_4_ continuously, like the Geostationary Carbon Cycle Observatory, Carbon Mapper, GHGSat, and the Copernicus Carbon Dioxide Monitoring, specific goals like wide swath, high spatial resolution, frequent revisit, and high accuracy can be anticipated. (3) Artificial intelligence and machine-learning methods can help dig into information that is not directly reflected in satellite imagery. For example, machine-learning methods have been used to automatically enhance the signals of CO_2_ measurements by satellites, improving the accuracy of inversion results,^1^ or identifies the operation of factories to track the operational status and emissions of these key point sources.

## Global satellite networking

The satellite constellation is a growing tendency for coming missions that will keep improving the monitoring through the increase in satellite numbers and technologies. Combine all available satellite observations to improve the spatial and temporal accuracy of near-real–time global emission map and assimilation models could help to finally realize global carbon emission monitoring.

An integrated observing system is essential to space-based measurements. Following the roadmap of the Committee on Earth Observation Satellites^10^ for integrating surface and airborne measurements to support the global stocktake, several carbon monitoring systems have been proposed, such as the CoCO_2_ project with the Copernicus Program (https://coco2-project.eu/, European Union) and the NASA Carbon Monitoring System (https://carbon.nasa.gov/, the USA), to accelerate the integration of multiple observation sources and to provide verifiable and transparent data products.

Given the large-scale data generated by satellites and integrated observing systems, standardization and sustainable reusing of observations are guaranteed for more efficient data use. The Copernicus Climate Change Service (https://climate.copernicus.eu/) has provided a landmark example of large-scale climate data sharing for a global data-exchange strategy and climate services. A free and open data-sharing platform led by governments will help break down the current data barriers caused by the monopoly of different stakeholders. Cloud-based platforms provide large-scale storage and supercomputing power, such as the Copernicus Climate Data Store toolbox (https://cds.climate.copernicus.eu/) or Google Earth Engine (https://code.earthengine.google.com/). The cloud-based platforms are needed to pool more available observation data and improve the computing power of the earth system modeling and inversion and assimilation system, to increase the spatial resolution from the current 0.1 × 0.1° to the kilometer level, and to shorten the temporal interval from annually to monthly or daily.

## Concluding remarks

Human activity data-based emission inventory can provide a sector-specific, systematic dataset that is comparable between countries, while satellite observations can support independent, low-cost, spatially distributed, and directly observed datasets, which are especially beneficial for areas that lack bottom-up data. Combining the superiorities of the near-real–time dataset and the satellite observations, an innovative technical route is proposed for transitioning from inventory-based carbon monitoring to satellite-driven carbon monitoring and from point-source carbon monitoring to the global scale. A near-real–time carbon map primarily derived from emission inventory with poor resolution in local areas can provide prior information for satellite observations and constrain satellite retrievals. Continuous point-source monitoring by satellites, in turn, can improve accuracy to form a dual-update mechanism with a long-term goal of quantifying anthropogenic emissions by satellite constellation only as an independent method. Based on this mechanism, gathering in-orbit and soon-to-be-launched satellites into a comprehensive observing network allows us to widen the monitoring space from a key point to a unified globe, jointly contributing to the data foundation for implementing international climate treaties and climate policies, and finally pave the way to a more accurate and transparent global stocktake.
